# Code-Switching Automatic Speech Recognition for Nursing Record Documentation: System Development and Evaluation

**DOI:** 10.2196/37562

**Published:** 2022-12-07

**Authors:** Shih-Yen Hou, Ya-Lun Wu, Kai-Ching Chen, Ting-An Chang, Yi-Min Hsu, Su-Jung Chuang, Ying Chang, Kai-Cheng Hsu

**Affiliations:** 1 Artificial Intelligence Center for Medical Diagnosis China Medical University Hospital Taichung City Taiwan

**Keywords:** nursing records, automatic speech recognition, code-switching, transfer learning, meta–transfer learning

## Abstract

**Background:**

Taiwan has insufficient nursing resources due to the high turnover rate of health care providers. Therefore, reducing the heavy workload of these employees is essential. Herein, speech transcription, which has various potential clinical applications, was employed for the documentation of nursing records. The requirement of including only one speaker per transcription facilitated data collection and system development. Moreover, authorization from patients was unnecessary.

**Objective:**

The aim of this study was to construct a speech recognition system for nursing records such that health care providers can complete nursing records without typing or with only a few edits.

**Methods:**

Nursing records in Taiwan are mainly written in Mandarin, with technical terms and abbreviations presented in both Mandarin and English. Therefore, the training set consisted of English code-switching information. Next, transfer learning (TL) and meta-TL (MTL) methods, which perform favorably in code-switching scenarios, were applied.

**Results:**

As of September 2021, the China Medical University Hospital Artificial Intelligence Speech (CMaiSpeech) data set was established by manually annotating approximately 100 hours of recordings from 525 speakers. The word error rate (WER) of the benchmark model of syllable-based TL was 29.54% in code-switching. The WER of the proposed model of syllable-based MTL was 22.20% in code-switching. The test set comprised 17,247 words. Moreover, in a clinical case, the proposed model of syllable-based MTL yielded a WER of 31.06% in code-switching. The clinical test set contained 1159 words.

**Conclusions:**

This paper has two main contributions. First, the CMaiSpeech data set—a Mandarin-English corpus—has been established. Health care providers in Taiwan are often compelled to use a mixture of Mandarin and English in nursing records. Second, an automatic speech recognition system for nursing record document conversion was proposed. The proposed system can shorten the work handover time and further reduce the workload of health care providers.

## Introduction

### Background

In Taiwan, more than 80% of health care providers are employed in the acute care system, mainly providing care for inpatients. However, the turnover rate of new recruits in medical centers is considerably high, exceeding 22.5%; in regional hospitals, it is approximately 29% [1-3]. Turnover rate is defined as the number of people leaving per month divided by the average number of people in the month. According to the equation below, Price [4] thinks that the turnover rate exceeds 50%, which is potentially harmful; Gauerke [5] considers that the ideal annual turnover rate should be approximately 5% to 10%. On average, 1 in 4 new health care providers leaves because they cannot endure the harsh working conditions in hospitals. Specifically, the reasons for the high turnover rate are presented as follows. First, the clinical workload is not divided into professional and nonprofessional work; thus, delegating nonprofessional work to others is not possible and, as such, nursing personnel are responsible for various menial tasks. Second, the particularity of three-shift work is highly inconvenient [6,7].



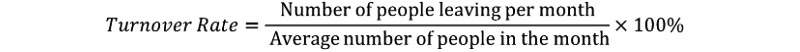



Health care providers spend most of their time in patient rooms, including isolation rooms, and on administrative tasks, including completing nursing records and charting work and reviewing them [8,9]. Descriptions of medical evaluations and their results are included in nursing records such that the medical care team can fully understand the patient’s condition. Incomplete or erroneous nursing records are a serious violation of patients’ rights. The causes of incomplete nursing records are inadequate human resources and differences in writing styles, which increase cognitive differences during health care providers’ shifts and can cause problems in patient care. According to the American Joint Commission International Accreditation Standards for Hospitals, approximately 65% of medical care problems are due to miscommunication [10]. Therefore, nursing records play a vital role in medical care.

Speech recognition programs, which have seen various advances in recent years, can understand human language; their application to the generation of nursing records can potentially improve work efficiency and reduce the workload of health care providers. Many countries have employed speech recognition technology in medicine [11,12]. The speech-to-text automation of nursing records can lighten the burden of administrative work. Although automatic speech recognition in the medical domain was first reported in the 1980s [13], all subsequent studies up to 1999 tested the transcription of single words as opposed to continuous speech in this context [14]. In recent years, a few studies have been conducted on speech recognition in the medical domain in terms of the word error rate (WER) [15-17].

### Related Work

The connectionist temporal classification (CTC) [18] and the listen, attend, and spell (LAS) [19] frameworks have been used to record physician-patient conversations [20]. The corpus size is 14,000 hours. Data cleaning comprised a major portion of the work. The WERs of the CTC and LAS methods were 20.1% and 18.3%, respectively, but both methods can be used to transcribe conversations between health care providers and patients. For the CTC method, a clean corpus and a corresponding language model are integral. Compared with the CTC method, the LAS framework has a higher tolerance to corpora containing a small amount of error. Because all the contextual information is considered by the LAS method, its accuracy is slightly higher than that of other models; however, the LAS method cannot perform streaming of automatic speech recognition. In addition, the length of the input voice segment has a significant impact on the accuracy of the model.

The learning method proposed in Winata et al [21] was designed to overcome the shortage of code-switching data by generating synthetic code-switching sentences. In that study, a sequence-to-sequence model was presented, and pointer-generator networks were used to generate code-switching data. The model had two principal characteristics: learning how to combine words from parallel sentences and determining when to switch from one language to another. However, the model was applied only to general conversations and not to conversations in a specific field. The main reason is that there are few conversations in the nursing data set, mainly nursing records, including records of patients’ physiological conditions, records of treatment and medication, and records of doctors’ orders.

### The Goal of This Study

The shortage of nursing labor in Taiwan has been a concern of Taiwan’s Ministry of Health and Welfare in recent years, and this situation has been exacerbated and highlighted by the spread of COVID-19 [22,23]. Health care providers in Taiwan have a heavier workload compared with those in other regions and countries. To mitigate this problem, we established a code-switching automatic speech recognition system enabling health care providers to complete nursing records without typing or editing. We used the China Medical University Hospital Artificial Intelligence Speech (CMaiSpeech) data set for this purpose.

## Methods

### CMaiSpeech Data Set

In the medical field, automatic speech recognition poses several difficulties. First, background noise is often present during recordings. Second, speaking speed varies considerably among individuals. Third, speech may contain hesitations and utterances, such as repeated words and restarted sentences. The most formidable challenge is code-switching. Taiwanese people often use a mixture of Chinese and English in daily conversation; this is often observed in the medical field and in nursing records. Medical terms, including thousands of drug names, are often written in English or represented by English abbreviations, meaning that sentences commonly contain a mix of Mandarin and English words.

**Figure 1:**
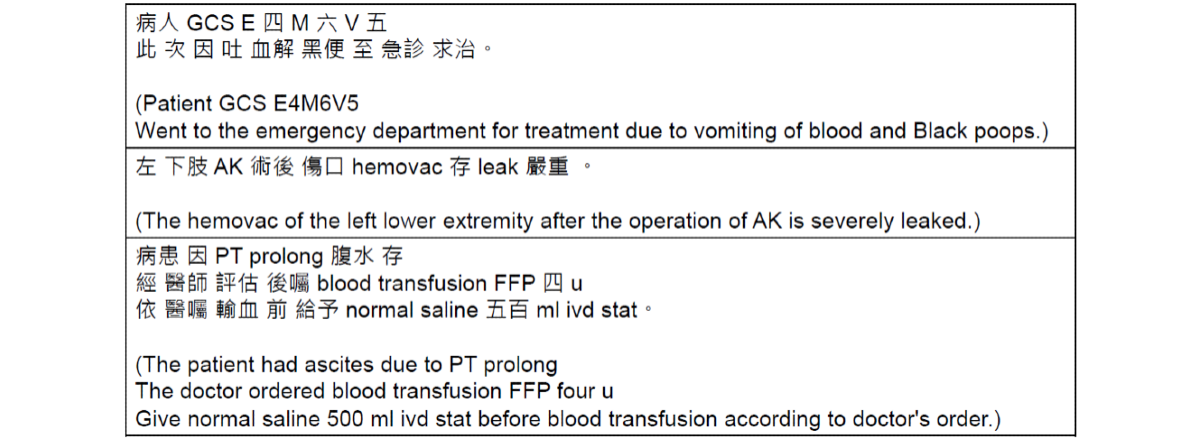
Examples of Mandarin-English code-switching utterances in clinical nursing records.

Nursing records in Taiwan mostly contain Mandarin-English sentences. To obtain a code-switching corpus for training the automatic speech recognition model, a nursing record corpus was created and used as the Mandarin-English code-switching corpus. The process of the CMaiSpeech data set has been created as follows. First, some code-switching sentences were selected from the nursing records in the China Medical University Hospital (CMUH) database. Next, volunteers were recruited to speak and record these sentences. After the recording process was completed, each sentence was annotated based on the recording context. Finally, the audio data were converted into a 16-kHz pulse-code modulation format. Figure 1 displays examples of Mandarin-English code-switching utterances in clinical nursing records.

Code-switching is a common language phenomenon and refers to a person alternately using more than one language or its variants in a conversation. A generalized definition of code-switching includes language alternation within sentences (ie, words of different languages are in the sentence) and outside the sentences (ie, sentences of other languages are in the data set). For our cases, most language alternations happened within sentences. The CMaiSpeech data set contains numerous medical terms in both English and Mandarin. The data set was established by manually annotating approximately 100 hours of recordings from 525 speakers. Most of the speakers were nurses employed at CMUH.

### Ethics Approval

This research was approved by the Institutional Review Board of China Medical University Hospital (CMUH), under approval No. CMUH110-REC2-187.

### National Education Radio Data Set

CMaiSpeech is a code-switching data set. But model training requires a Mandarin corpus with a Taiwanese accent. Therefore, National Education Radio (NER) content is considered a Mandarin data set. NER content from the Formosa Speech in the Wild data set [24] is a Mandarin corpus. For the Mandarin corpus, volume 1 of the NER data set, which contains approximately 100 hours of speech recordings, was used. The data were split into training and evaluation sets by the setup described in the source (ie, volume 1 of the NER data set). Both CMaiSpeech and NER data sets were employed in model training, validation, and testing.

### Transfer Learning

Transfer learning (TL) refers to the transfer of a trained model and parameters to a new model such that the new model does not need to be retrained [25]: this is considered positive transfer. In 2020, Joshi et al [26] applied TL to the recurrent neural network transducer model in the field of speech recognition [27,28]. When the learning of one kind of knowledge promotes the learning of another kind of knowledge, it is called positive transfer. On the contrary, when the learning of one kind of knowledge hinders the learning of another kind of knowledge, it is called negative transfer. For example, when the tasks of the source domain and the target domain are not related, this may lead to negative transfer. In this study, the TL model was regarded as a benchmark model, and the CMaiSpeech and NER data sets were used for two-stage training.

### Meta–Transfer Learning

Meta-TL (MTL), proposed in Winata et al [29], is used to overcome the challenges encountered in code-switching speech recognition. Specifically, this model is used to map audio recordings to graphemes with relatively few resources. MTL is based on model-agnostic meta-learning [30], with a few modifications for the code-switching tasks. These modifications exchange the model’s ability to perform a wide range of tasks for a more favorable ability to initialize code-switching tasks.

### Model Selection

**Figure 2:**
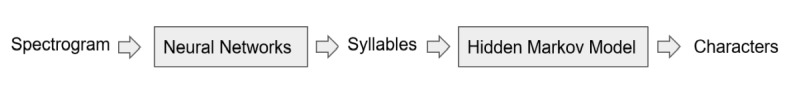
The difference between syllable-based and character-based models.

The mainstream automatic speech recognition system can be divided into two models: syllable based and character based. Figure 2 shows the difference between the two models. The character-based model uses the hidden Markov model (HMM) to convert syllables to characters. On the contrary, the syllable-based model has no converter. In Taiwan, nursing records are mainly written in Mandarin, with technical terms and abbreviations being written in both Mandarin and English. Accordingly, we selected a method suitable for nursing handover. The syllable-based method [30] is used in Mandarin, wherein Chinese words are converted into syllables to reduce their complexity. Next, a cascade syllable decoder is used to learn the lingual information to map syllables to graphemes.

Although the most commonly used Chinese transliteration system in Taiwan is Bopomofo [31], we employed simplified pinyin (ie, standard Chinese pinyin without tone markers) to standardize the form of the English and Mandarin words at the character level. Task complexity was reduced through vocabulary minimization. For syllable decoding, the HMM was used along with the Viterbi algorithm. A lexicon containing English words was required to decode syllables produced by the speech transformer model into graphemes.

**Figure 3:**
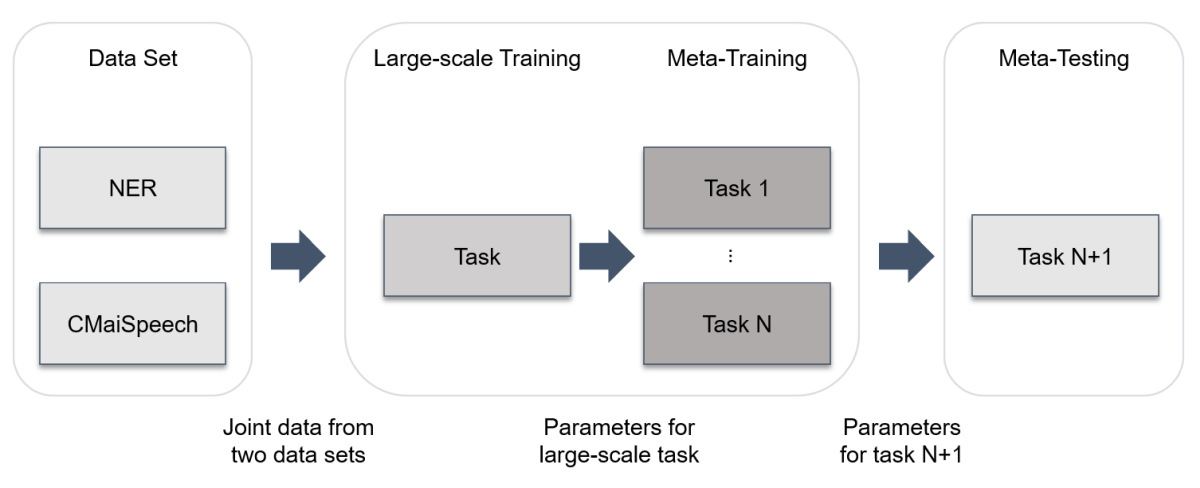
Training and testing flowchart of the meta-transfer learning models. NER: National Education Radio; CMaiSpeech: China Medical University Hospital Artificial Intelligence Speech.

The training and testing flowchart of the proposed MTL models is shown in Figure 3. The training and testing flowchart of the MTL models is made up of three blocks. First, regarding the data set block, the monolingual data set (ie, NER) and code-switching data set (ie, CMaiSpeech) were jointly learned through meta-training; the corpora of the CMaiSpeech and NER data sets were converted to characters or syllables, a step that can be understood as audio conversion into features; and character-based and syllable-based models were built. Second, regarding the training block, the models were trained using the MTL method proposed by Guzmán et al [15]; all models can then be further fine-tuned. In addition, for the syllable-based model, an HMM was trained using the CMaiSpeech data set and a lexicon that maps Mandarin characters to pinyin. Third, regarding the testing block, meta-testing was used.

## Results

### Data Set Analysis

The MTL and TL algorithms were evaluated and compared with the CMaiSpeech and NER data sets. The statistics and experimental settings of the data sets are presented in Table 1. Regarding wave statistics, approximately 126 hours of monolingual Mandarin data (ie, NER data set) and approximately 107 hours of code-switching data (ie, CMaiSpeech data set) were employed for training and validation, respectively. Next, the TL and MTL models were assessed using approximately 1.8 hours of code-switching data (ie, CMaiSpeech data set). The CMaiSpeech data set contained 136,196 English words and 787,701 Chinese words, whereas the NER data set contained 682 English words and 1,420,101 Chinese words. On the basis of the proportions of English and Chinese words, the code-switching level of the CMaiSpeech data set was substantially higher than that of the NER data set. Therefore, the CMaiSpeech data set was determined to be more useful for testing. The testing corpus of the CMaiSpeech data set contained 2427 English words and 14,820 Chinese words. To quantify the code-switching level, the language entropy was used to evaluate the code-switching level. The language entropy of the CMaiSpeech and NER data sets in the training process was 0.6033 and 0.0060, respectively, and the language entropy of the CMaiSpeech data set in the testing process was 0.5857. The probability of switching (ie, the integration index [I-index]) [30] was used to describe the code-switching frequency. The mean I-indexes of the CMaiSpeech and NER data sets in the training process were 0.2006 and 0.0006, respectively, and the mean I-index of the CMaiSpeech data set in the testing process was 0.1411. The frequency of Chinese and English words in the CMaiSpeech data set was determined (Multimedia Appendix 1).

**Table 1:** Statistics and experimental setting of the CMaiSpeech and NER data sets.

Methods			CMaiSpeech^a^ data set				NER^b^ data set					CMaiSpeech data set
			Training		Validation		Training		Validation		Testing	
**Wave statistics**												
	Duration, hours	105.5334		1.6572		114.2523		12.4390		1.8093		
	Mean duration, seconds	14.2634		18.9395		21.6751		21.2028		17.5566		
	Utterances, n	26,636		315		18,976		2112		371		
**Word statistics, n**												
	English words	136,196		2342		682		150		2427		
	Chinese words	787,701		12,115		1,420,101		206,909		14,820		
	Total words	923,897		14,457		1,420,784		207,059		17,247		
**Code-switching level**												
	Language entropy	0.6033		0.6391		0.006		0.0086		0.5861		
	I-index^c^, mean (SD)	0.2006 (0.1529)		0.1896 (0.16)		0.0006 (0.0052)		0.0011 (0.008)		0.1411 (0.1372)		

^a^CMaiSpeech: China Medical University Hospital Artificial Intelligence Speech.

^b^NER: National Education Radio.

^c^I-index: integration index (ie, the probability of switching).

### Model Analysis

Table 2 lists the performance of various automatic speech recognition models on CMaiSpeech test sets in WERs. The benchmark models included the character-based TL model, the syllable-based TL model, and the Microsoft Azure cloud application programming interface (API) [32]. The proposed models were the character-based MTL model and the syllable-based MTL model. The total number of words in the testing set was 17,247. The output results were Chinese characters and English words. The test sets had two characteristics: the sentences code-switched between English and Mandarin and the sentences included many drug names and English abbreviations.

Among the evaluated models, the WERs of the character- and syllable-based TL models were 27.22% and 29.54% WER–code-switching (WER-CS), respectively, whereas that of the Azure cloud API was 59.20% WER-CS. By contrast, the WERs of the proposed character- and syllable-based MTL models were 24.78% and 22.20% WER-CS, respectively. In most cases, the MTL models yielded higher WER values than did the TL models because the CMaiSpeech data set was smaller. The MTL algorithm is suitable for solving small-sample learning problems because the MTL model was jointly trained using both the CMaiSpeech data set and the NER data set. By contrast, the TL algorithm is usually pretrained on a data set to obtain a more favorable initial model, which is then fine-tuned on the new data set. The new data set must have a considerable amount of data; this is typically not the case in a small-sample learning task. Thus, joint training enables the MTL model to have robust code-switching capabilities.

For the same test sets, the performance of syllable-based automatic speech recognition models without HMM was measured on CMaiSpeech test sets (Table S1 in Multimedia Appendix 2). The WERs of the syllable-based TL and syllable-based MTL models without HMM were 24.16% and 16.85% WER-CS, respectively. The output of the models was syllables, not Chinese characters and English words.

**Table 2:** Performance of various automatic speech recognition models on CMaiSpeech^a^ test sets (code-switching) in terms of the word error rate.

Methods	WER-CS^b^, %	WER-EN^c^, %	WER-ZH^d^, %
Character-based transfer learning	27.22	40.15	26.75
Syllable-based transfer learning	29.54	38.13	29.22
Azure cloud application programming interface	59.20	95.57	54.23
Character-based meta–transfer learning (proposed)	24.78	41.23	24.28
Syllable-based meta–transfer learning (proposed)	22.20	36.13	21.54

^a^CMaiSpeech: China Medical University Hospital Artificial Intelligence Speech.

^b^WER-CS: word error rate–code-switching.

^c^WER-EN: word error rate–English.

^d^WER-ZH: word error rate–Zhōngwén.

### External Verification

Two experiments were conducted for external verification. First, to analyze the performance of the proposed models and the public automatic speech recognition system, based on the experimental settings, the same CMaiSpeech test sets were applied to the automatic speech recognition model of the Azure cloud API. The WER of the Azure cloud API was 59.20% WER-CS (Table 2). This may be attributable to the fact that the CMaiSpeech test sets included domain-specific conversations about nursing care, whereas the Azure cloud API mainly included general conversations.

Second, the proposed models were deployed online in the Medical Information Technology Office of CMUH. The clinical handover data were collected and used as clinical test sets to validate various automatic speech recognition models (Table 3). The total number of words in the clinical test sets was 1159. The WER of the syllable-based TL models was 33.65% WER-CS, whereas that of the Azure cloud API was 31.75% WER-CS. As for the proposed syllable-based MTL model, the WER was 31.06% WER-CS. The WERs of the proposed systems from the online results were not as favorable as those from the offline results, possibly because the clinical test sets contained a high proportion of Mandarin words and a low proportion of English words. Therefore, when output English words were misspelled, their WERs were substantially higher than those of the Chinese words. In the future, we must focus on increasing the size of the training database to optimize the performance of the proposed automatic speech recognition engine.

Interesting experimental results were found, as seen in Table 3. If the test sets were divided into a long sentence part and a short sentence part, with 15 seconds as the boundary, the length was categorized and the WERs were calculated separately, as shown in Table 4.

**Figure 4:**
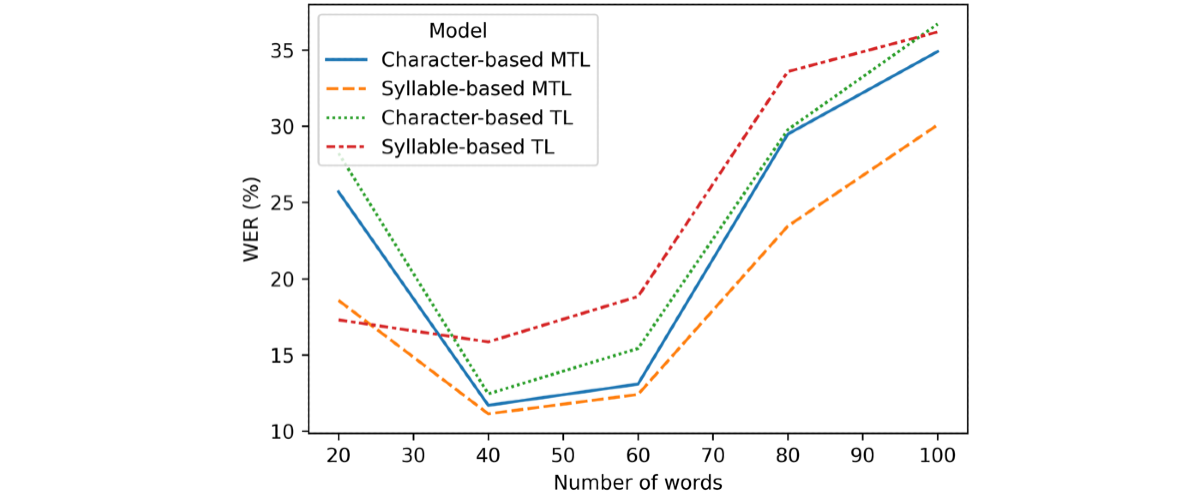
The relationship between sentence length and the word error rate (WER). MTL: meta–transfer learning; TL: transfer learning.

Figure 4 shows the relationship between sentence length and the WER. From the experimental results, it can be found that the sentence length has an inflection point at 40 words. When the sentence length is more than 40 words, the sentence length is positively correlated with the WER, and this result is understandable. On the contrary, when the sentence length is less than 40 words, the sentence length is negatively correlated with the WER. There are two main reasons for this. First, uncommon words in short sentences increase the probability of identification errors. Second, there are limited previous occurrences that can be referenced. Both of these reasons may increase the WER value. In addition, the relationship between the code-switching level and the WER was determined (Multimedia Appendix 3). From the experimental results, it can be found that the code-switching level has an inflection point at 0.40. When the code-switching level is more than 0.40, the code-switching level is positively correlated with the WER.

**Table 3:** Performance of the benchmark and proposed models on clinical test sets in the clinical field in terms of the word error rate.

Methods	WER-CS^a^, %	WER-EN^b^, %	WER-ZH^c^, %
Character-based transfer learning	60.40	107.59	56.88
Syllable-based transfer learning	33.65	85.58	30.31
Azure cloud application programming interface	31.75	96.08	27.80
Character-based meta–transfer learning (proposed)	43.57	114.06	38.41
Syllable-based meta–transfer learning (proposed)	31.06	89.52	27.84

^a^WER-CS: word error rate–code-switching.

^b^WER-EN: word error rate–English.

^c^WER-ZH: word error rate–Zhōngwén.

**Table 4:** Performance of the benchmark and proposed models on long and short sentences in terms of the word error rate.

Methods	Long sentences, WER^a^, %	Short sentences, WER, %
Character-based transfer learning	33.58	10.38
Syllable-based transfer learning	35.55	13.63
Character-based meta–transfer learning (proposed)	30.87	8.66
Syllable-based meta–transfer learning (proposed)	27.14	9.11

^a^WER: word error rate.

## Discussion

In this study, a Mandarin-English code-switching speech recognition system was developed using a corpus of nursing records to allow such records to be completed without typing or editing. The proposed system can lighten the heavy workloads of medical personnel. The WER of the benchmark syllable-based TL model was 29.54% WER-CS, and that of the proposed syllable-based MTL model was 22.20% WER-CS. The test sets comprised 17,247 words. In a real clinical case, the WER of the proposed model was 31.06% WER-CS. The clinical test sets contained 1159 words.

Future studies should focus on training with large Mandarin corpora to improve the performance of the proposed code-switching speech recognition engine. We plan to release the proposed corpus of nursing data with the accompanying processing software to the research and development community for the study of clinical language processing.

## Appendix

Multimedia Appendix 1 Frequency of Chinese and English words in the CMaiSpeech data set. CMaiSpeech: China Medical University Hospital Artificial Intelligence Speech.

Multimedia Appendix 2 Performance of syllable-based automatic speech recognition models without the hidden Markov model (HMM) on the CMaiSpeech test set. CMaiSpeech: China Medical University Hospital Artificial Intelligence Speech.

Multimedia Appendix 3 The relationship between the code-switching level and the word error rate (WER). I-index: integration index; MTL: meta–transfer learning; TL: transfer learning.

## Abbreviations

API: application programming interface
CMaiSpeech: China Medical University Hospital Artificial Intelligence Speech
CMUH: China Medical University Hospital
CTC: connectionist temporal classification
HMM: hidden Markov model
I-index: integration index
LAS: listen, attend, and spell
MTL: meta–transfer learning
NER: National Education Radio
TL: transfer learning
WER: word error rate
WER-CS: word error rate–code-switching
