# Supplementary figures and images for: Code-Switching Automatic Speech Recognition for Nursing Record Documentation: System Development and Evaluation
Source: JMIR Nurs. 2022 Dec 7;5(1):e37562. doi: 10.2196/37562 (PMC9773023; doi:10.2196/37562)

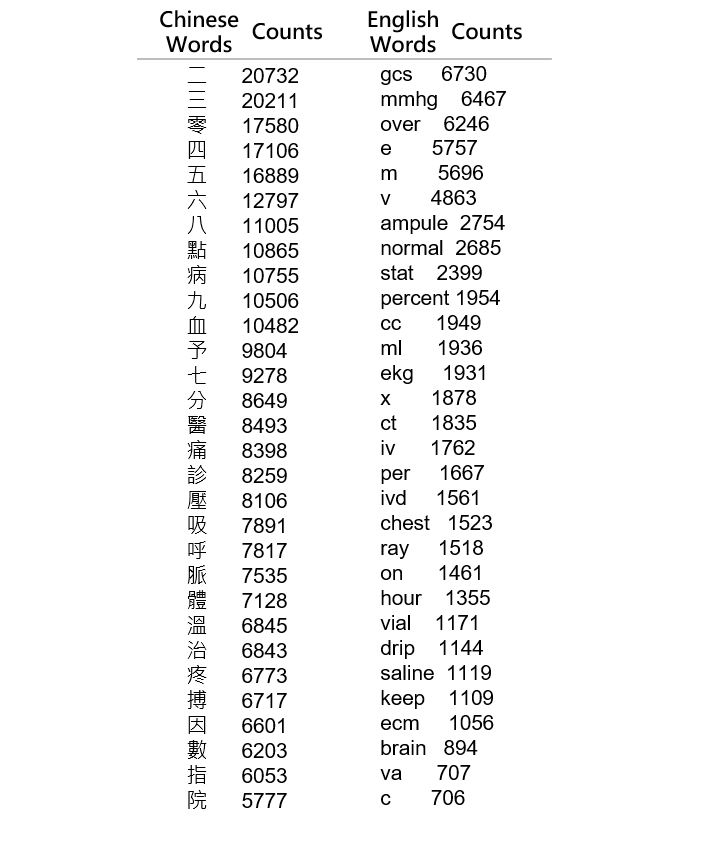

Supplement: Multimedia Appendix 1 [file nursing_v5i1e37562_app1.png]

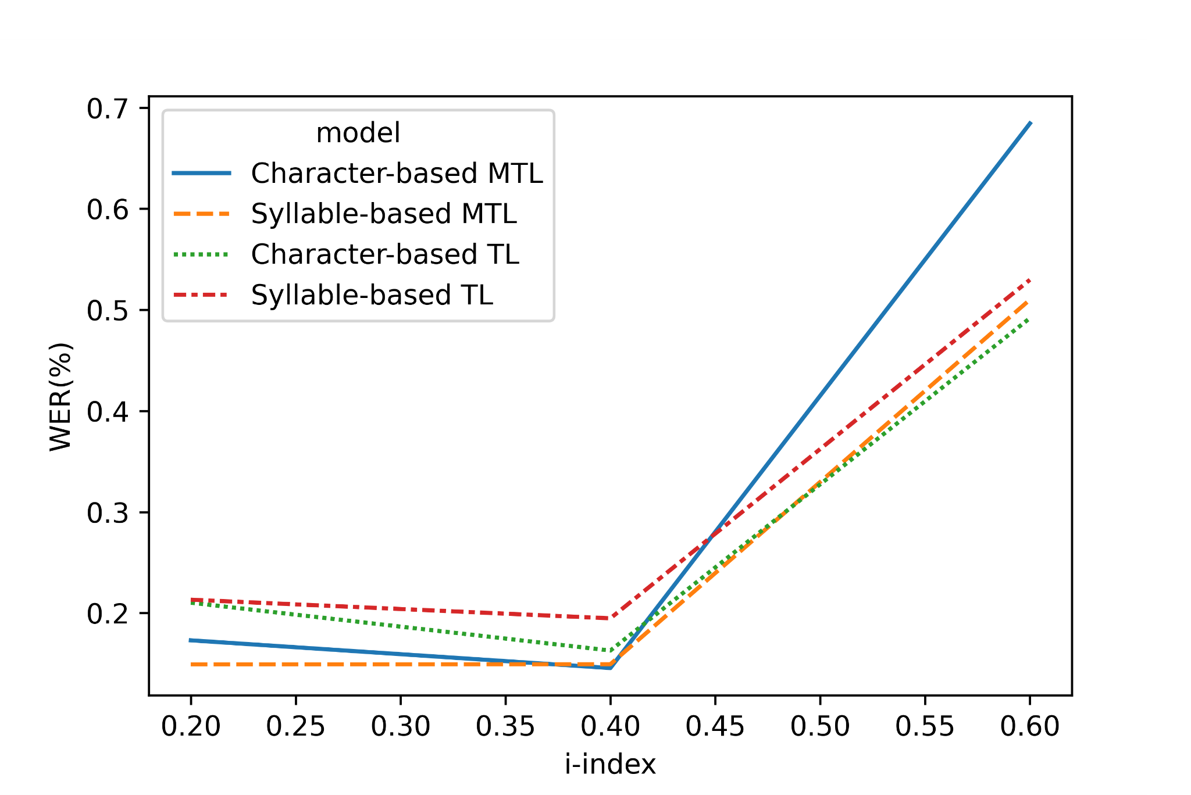

Supplement: Multimedia Appendix 3 [file nursing_v5i1e37562_app3.png]
