# Supplementary material for: Code-Switching Automatic Speech Recognition for Nursing Record Documentation: System Development and Evaluation
Source: JMIR Nurs. 2022 Dec 7;5(1):e37562. doi: 10.2196/37562 (PMC9773023; doi:10.2196/37562)
Supplement: Multimedia Appendix 2 [file nursing_v5i1e37562_app2.docx]

**Multimedia Appendix 2**

**Table S1.** Performance of syllable-based automatic speech recognition models without HMM on CMaiSpeech test sets (code-switching) in terms of word error rate.

| **Methods** | **WER-CS (%)** | **WER-EN (%)** | **WER-ZH (%)** |
| --- | --- | --- | --- |
| Syllables-based TL without HMM | 24.16 | 24.16 | 0 |
| Proposed Syllables-based MTL without HMM | 16.85 | 16.85 | 0 |
